# Addressing Workforce Challenges with an Apprenticeship-Based Training Program for Paraprofessionals in Behavioral Health: Conceptual Framework and Effectiveness

**DOI:** 10.3390/bs16030441

**Published:** 2026-03-17

**Authors:** Nicholas D. Mian, Macey Muller, Erin Singer, Hannah Lessels, Jen Williams, JoAnne Malloy

**Affiliations:** 1 Department of Life Sciences, College of Professional Studies, University of New Hampshire, Manchester, NH 03101, USA; 2 Institute on Disability, University of New Hampshire, Durham, NH 03824, USA; macey.muller@unh.edu (M.M.); joanne.malloy@unh.edu (J.M.); 3 JSI Research & Training Institute, Inc., 44 Farnsworth St #7, Boston, MA 02210, USA; erin.singer@jsi.org (E.S.); hannah.lessels@jsi.org (H.L.)

**Keywords:** behavioral health, paraprofessional, apprenticeship, peer recovery specialist, workforce, substance use disorder

## Abstract

There is a need to enhance the behavioral health (BH) workforce. Paraprofessionals and peers are often on the “front lines” working with families affected by substance misuse. While they possess valuable lived experience, they often lack the requisite education to be most effective, resulting in high burnout and turnover. This study describes a novel training program for paraprofessionals working in family BH that included three online, 8-week courses (Level I) and a 12-month supervised apprenticeship (Level II). This study measured program satisfaction and effectiveness (knowledge, confidence, and perceived competence) and explored effects on career intention. A sample of paraprofessionals in the BH workforce provided data at baseline, after Level I, and after Level II. After Level II, 87% of participants rated their satisfaction with the program as high. Statistically significant improvements were found for knowledge, confidence, and competence across all domains. Almost all participants reported increased confidence after each level (93% and 94%, respectively). The majority (69%) reported increased interest in continuing their BH career and education. Overall, results suggest that the program was well-received by participants and was associated with improvements. Results provide preliminary support for apprenticeship-based training to enhance the BH workforce and address workforce challenges.

## 1. Introduction

### 1.1. Behavioral Health Challenges

The United States continues to experience a severe opioid crisis, especially in the most impoverished regions. There are clear links between poverty and substance misuse (Daley, 2013; Hedegaard et al., 2017). Children of parents who have opioid use disorders or other substance use disorders (SUDs) are at increased risk for abuse and neglect, and for developing academic, emotional, social, and behavioral impairments (Ashrafioun et al., 2011; Winstanley & Stover, 2019). Research shows there may be long-term developmental harm among children and youth who were exposed to opioids in utero, including learning problems, attention deficit-related disorders, and behavioral problems (Fill et al., 2018; Yen & Davis, 2022).

There are diverse and complex social dimensions to engaging families with parental substance misuse in services and supports, not the least of which are stigma, economic burdens, emotional distress, and the intense recovery work that is required to help the individual and the family (Daley, 2013). Due to the complex and dynamic nature of their needs, along with the fact that many children of substance-misusing parents are in continuous crisis, these families require holistic, individualized, interagency, and comprehensive support.

As the country continues to experience behavioral health (BH) needs, a current workforce shortage only exacerbates this challenge (Beck et al., 2018). While there is a significant interest among bachelor’s level students in the mental health field, many students are “opting out” of this field altogether, mostly due to perceived economic barriers (Mian & Glutting, 2025). Many more bachelor’s-level workers—many of whom have significant educational debt—would likely go into this field if they were offered more competitive salaries. Economic barriers exist at the graduate level as well, where graduate tuition in the mental health fields has grown to levels that make graduate study unattainable for many (Wilcox et al., 2021). Hence, economic factors represent significant barriers to ensuring that the BH workforce can meet the needs. Even the present BH workforce is under pressure—high caseloads, increased severity of need, burdensome documentation requirements, and low reimbursement and pay rates all contribute to high turnover rates and burnout (Ballout, 2025).

### 1.2. The Role of Paraprofessional Workers and Inherent Challenges

Paraprofessionals are trained individuals who assist or work under the supervision of a professional and work in positions that do not require an advanced degree. Paraprofessionals in BH represent diverse roles, including school support staff, family support specialists, community health workers, and peer support specialists who use their own lived experience to support individuals and families. While paraprofessionals generally lack higher education degrees, they show great promise as part of a solution to workforce challenges in BH care (Barnett et al., 2018; Kanzler et al., 2024). Peer support workers—known by many terms, including peer recovery specialists—are individuals who have undergone similar experiences, are in recovery themselves, and are thereby uniquely qualified to provide hope, advice, validation, and social support to individuals in various stages of behavioral health recovery (Lowder et al., 2025). Paraprofessionals occupy an important area in the BH workforce, especially since significant changes in US policies allow for Medicaid (healthcare coverage for low-income individuals) reimbursement for a wider array of services in some states. Most notably, Certified Recovery Support Workers and peer recovery coaches/support specialists have become a critical piece of the substance use treatment and recovery field because of their ability to effectively engage and connect with clients (Gagne et al., 2018; Satinsky et al., 2020). Other paraprofessionals in BH, such as family support workers, work directly with families affected by SUD or mental illness and are involved in a vast range of activities to support individuals and families (Gagne et al., 2018). Paraprofessionals are also employed widely in the Defense Health Agency and the Veteran’s Healthcare Administration in the treatment of substance misuse (Kanzler et al., 2024). Community Health Workers are already employed in these capacities in many other countries and have a strong record of delivering evidence-based interventions (Barnett et al., 2018). While recognized for these important roles, paraprofessionals also represent an important pathway into more advanced levels of the BH workforce. These individuals are often driven to work in the BH field due to their own lived experiences or what they have seen and experienced in their communities.

The most significant limitation of the paraprofessional workforce is the lack of required or standardized training and focused supervision (Areán et al., 2025; Bipartisan Policy Center, 2023; Kanzler et al., 2024), which can lead to several types of barriers. These barriers can affect their on-the-job work performance and ability to thrive in a professional setting, including: (1) a lack of training in SUD and evidence-based interventions, potentially reducing efficacy in their work with clients, (2) difficulties with self-care to address challenges that are inherent to the BH field, such as vicarious trauma, and (3) lack of training in cultural awareness and sensitivity to diversity, which can limit client success. Lack of training and ongoing supervision has been shown to have a negative impact on an individual’s knowledge, skills, attitude and overall quality of treatment (Bina et al., 2008). Further, other barriers reflect problems with professional development, including: (1) limited awareness and perceived accessibility of opportunities for job growth and higher education opportunities, (2) perceived prejudice/low credibility from other workers in the BH field (Kanzler et al., 2024), and (3) low salaries, which reduce opportunities for further education and training outside of full-time employment (Cooper et al., 2024; Gagne et al., 2018). There are some common skills that all paraprofessionals need regardless of service sector such as engaging clients, facilitating referrals and access to services and supports, and participating effectively on interdisciplinary teams. Given that they also work in various healthcare disciplines, each paraprofessional worker also needs training regarding the needs and services of their population and hands-on training regarding their workplace-specific responsibilities.

Taken together, the least prepared and often most poorly paid or supported members of the BH workforce routinely work with very challenging clients under challenging circumstances, explaining why burnout and turnover are so common. One review identified that the most salient aspect of burnout in mental health careers is emotional exhaustion—feeling unable to keep up with the emotional demands of the work (Paris & Hoge, 2010). One recent concept analysis of over 500 studies of job satisfaction in the nursing field found that an important aspect of satisfaction is self-efficacy, one’s belief in one’s own achievement potential (Heier & Nelson-Brantley, 2024). Logically, workers who lack appropriate training are more likely to experience challenges in workplace achievement. Unlike other identified factors, such as perceived meaningfulness of the work (Heier & Nelson-Brantley, 2024)—which most BH workers intrinsically possess—self-efficacy can be improved through informational and skills-based training. This same study also found that job satisfaction was associated with a higher level of commitment to one’s work (i.e., reduced turnover; Heier & Nelson-Brantley, 2024). These results suggest that increasing self-efficacy through training would be expected to positively affect commitment and reduce burnout and turnover.

### 1.3. Training Paraprofessionals in Behavioral Health Service Delivery

One potential solution to these workforce challenges is to increase reliance on paraprofessional-level staff but offer appropriate training specific to the workplace context and job duties (Areán et al., 2025; Bipartisan Policy Center, 2023). Paraprofessional workers can provide direct support services to families as well as certain skills-based interventions such as crisis intervention, implementing coping skills, and helping clients connect with community resources, while under the supervision of a licensed professional—evidence suggests paraprofessionals can perform these duties with success (Anvari et al., 2023; Barnett et al., 2018; Cooper et al., 2024; Kanzler et al., 2024; Satinsky et al., 2020). One recent study trained paraprofessionals to deliver evidence-based prevention programming in schools—results suggested the program was well-received by participating students and had a positive effect on student mental health (Bruns et al., 2025). In another example, paraprofessional-level peer recovery coaches were trained to deliver behavioral activation—an evidence-based intervention for depression—with good success (Satinsky et al., 2020). One meta-analysis of peer support approaches found evidence of efficacy for several conditions, including depression, self-efficacy, and recovery (Cooper et al., 2024). This same study found evidence of training and supervision as factors that were related to program success.

Several studies have suggested that training programs have been recommended as an intervention to reduce burnout and turnover among paraprofessionals (Paris & Hoge, 2010). In one study, training in SUD was predictive of a more positive therapeutic attitude towards SUD clients and feeling more prepared and competent (Bina et al., 2008). Further, such training programs represent a pathway through which paraprofessional-level workers may become academically and professionally prepared for ongoing education to fill much-needed positions at the bachelor’s and graduate levels.

The studies cited above provide evidence of paraprofessional-delivered interventions focused on the effects on patients/clients and the success of the intervention itself rather than the effects of the training on the paraprofessional trainees. We know very little regarding the effects that various types of training can have on the paraprofessionals’ perceived efficacy or career trajectory, for example. Community-based training models that go beyond didactics may be especially important in BH due to the high-touch and emotionally taxing nature of the work. Of course, direct experience with clients, in addition to coursework, is required for all advanced licensure in mental health and BH fields.

As previously noted, paraprofessionals have the potential to enhance the BH workforce (Bipartisan Policy Center, 2023). But before such a pipeline can be developed, there is a need to understand the experience of paraprofessionals participating in BH training.

### 1.4. The Apprenticeship Training Model

There has been a growing need to find alternative ways to properly train and support behavioral health workers, including developing feasible options for individuals to enter the workforce without having to make large upfront investments in formal academic programs. Apprenticeship programs offer a feasible, comprehensive, and effective option for training a wide swath of the workforce, but we do not know of any study that has investigated the efficacy of an apprenticeship model for training paraprofessionals in BH. Apprenticeship programs pair learners with mentors in the field to provide on-the-job training that is supplemental to coursework. In general, apprenticeship programs have been shown to improve employment outcomes, academic performance, and later higher education pursuit in vocational high school students (de Amesti & Claro, 2021). A recent meta-analysis found that apprenticeship occupations are being expanded to include health, education and personal care occupations, meeting a major workforce need in many communities (Butrica et al., 2023). All apprenticeship programs have common components, although they can vary in length, intensity, and organizational features. All programs include a formal training component, called related instruction, where trainees learn foundational theory and skills related to that occupation. This training is often provided by a community college, technical school, or private institution. Second, the hallmark of apprenticeship is the on-the-job, paid employment component. Apprentices experience hands-on learning in the occupation with expert mentorship, supervision, and performance feedback. The critical factor is that the apprentice is paid wages (often lower than the rate paid to experienced workers) while in a real-world work setting. On-the-job, hands-on experiences specific to the employment setting allow for skill development and are known to be the most effective way to sustain and enhance learning and performance. The benefits of traditional apprenticeship models have been well documented. Employers can benefit from apprenticeship in several ways, including the opportunity to “try out” potential employees, reduced hiring costs, the value of related instruction that can be costly for employers to provide, and the support received from the Department of Labor (DOL) and other program partners (Kuehn et al., 2022; Marotta et al., 2022). The apprentice can benefit from the opportunity to experience the specific requirements of the occupation as a paid employee and skills developed from classroom instruction and on-the-job mentoring (Reed et al., 2012). Apprentices who complete a formal DOL program also receive a credential that is recognized nationally and affirms that the worker has the skills to perform the duties of that occupation. Research also shows that apprenticeship programs reduce the varied costs to society of unemployment, especially among people who have difficulty finding jobs through traditional high school or college pathways (Manzo et al., 2019). In effect, apprenticeships offer a viable and organized career pathway for workers who may otherwise be left out of our economy.

Most of the research has been conducted with programs that have been traditionally associated with apprenticeships, such as construction, welding, and auto repair, among others. The program featured here is unique as it was one of the first approved DOL apprenticeships focused on a human services occupation.

### 1.5. Present Study

The present study sought to address one approach to BH workforce challenges by developing a training program for paraprofessionals and peers supporting children and families impacted by substance misuse. The conceptual framework of the program is based on the claim that burnout and associated problems among paraprofessional BH workers are in part due to a lack of training. If such training can improve areas such as knowledge, confidence, and competence, workers would be expected to feel more successful in their work, stay in their positions longer, and seek growth within their field of expertise rather than leave it. Further, such a training program would need to be well-received by participating workers. The present study focuses on specific aspects of this conceptual framework: how participation in a training program was experienced by and affected the paraprofessional workers.

The program we developed, called Building Futures Together (BFT), included three 8-week online courses, followed by 12 months of on-the-job registered apprenticeship with an assigned mentor in the field. We measured satisfaction, career intentions, and effectiveness (i.e., perceived knowledge, confidence, and competence). We made the following hypotheses: (1) participants would evidence high levels of satisfaction, and (2) program effectiveness would be evidenced by improvements in knowledge, confidence, and competence. We measured changes in career intentions as an exploratory area.

## 2. Materials and Methods

### 2.1. Program Description

The BFT program was funded by the Health Resources Services Administrations’ (HRSA) Opioid-Impacted Family Support Program, a funding agency of the US federal government (Heath Resources Services Administration, 2020), specifically to train paraprofessional-level staff to work with families, children, and youth impacted by substance use disorders, applying a U.S. Department of Labor Apprenticeship training model. HRSA also required grantees to allocate prescribed amounts of the grant to be awarded to trainees as stipends, with no constraints or restrictions on how the stipends could be used by the trainees, in full recognition that the trainees would be working full-time while trying to complete a rigorous training program and that most would be earning low wages.

We used this grant opportunity to address acute, local workforce challenges in behavioral health care. These challenges include vacancies and turnover rates that are especially high among family support workers. To address these needs, the BFT program strives to support each learner, build skills and knowledge, and help them advance in the field. The program was designed to meet US Department of Labor apprenticeship program standards for Human Services Assistants, the first such program in the country to our knowledge. The training consisted of two levels. Level I included 24 weeks of didactic learning via three online, asynchronous eight-week, college-level courses, including: (1) Certified Recovery Support Worker training; (2) Child Development and Family Systems; and (3) Enhanced Care Coordination. Courses two and three were administered through the learning management system of a state university in the northeast; course one was administered through a partnering community college. Efforts were made to adhere to Universal Design for Learning principles, which include providing instruction in multiple formats, using videos and other media to provide instruction, and allowing trainees multiple ways to demonstrate competency. Successful completion of Level I was considered a major milestone. Participants were invited to an awards ceremony in which they received a certificate of completion. They also earned a digital badge for their courses and received the academic qualifications to apply for a Certified Recovery Support Worker certification. Many considered this a major success regardless of continuing into Level II.

While not a requirement, eligible participants were given the opportunity to continue into Level II (the funding agency required only 70% of trainees to continue). Level II included 2000 h (generally, 1 year full-time) of on-the-job training at the trainees’ place of work, supported by a mentor. Mentors were selected by program staff and in most cases worked at the same agency as the participant.

At the completion of specific benchmarks, the trainee received a stipend, including $5000 during Level I and $7500 during Level II. Trainees also had the opportunity to receive a certificate of completion from the Department of Labor if they completed the training through an employer-registered apprenticeship site. College transfer credit was also available for trainees who were interested in continuing their education in the states’ community college or university system.

In addition to the stipends, the major costs required for the BFT program were personnel-based. The project included a full-time Senior Project Coordinator, a Project Coordinator, a Principal Investigator, and a Co-Principal Investigator. The program also included course Instructors who were compensated on a per-course basis. Mentors were compensated for time completing competency assessments. The team also consisted of an external evaluator with experience in program evaluation.

A key aspect of the BFT program was trainee support offered by Program Coordinators to improve retention and completion. The first and second cohorts had only one Masters-Level Project Coordinator who managed the day-to-day requests from participants and employers. This Project Coordinator had experience working with students on academic probation and those with learning disabilities in higher education through academic coaching with an academic comprehensive support program. The Project Coordinator’s experience and expertise helped support checking in on the trainees weekly through the online learning platform and being available to answer any questions or concerns, which allowed for a supportive relationship with participants. The team received additional funding to allow for a second, bachelor’s-level Project Coordinator to support trainees as needed. Both had an active role in Level I—similar to the role of a Teaching Assistant—through additional responses on discussion boards and follow-up when/if assignments were not handed in.

Consistent with evidence-based student coaching (Bettinger & Baker, 2014), the program included academic support on using the learning management system, advice on time management, help with self-advocacy, and support with course assignments. Assistance also included career support such as finding employment sites, mediating between instructors, mentors, employers, and other trainees, and helping problem-solve personal challenges. Program coordinators also offered several Saturday Seminars during Level I training, delivered via synchronous online meetings. These seminars were designed to create a learning collaborative with trainees to discuss course curriculum, share case studies on how they applied the new knowledge and skills to their everyday roles, and share community resources with each other. The seminars and online discussion boards created connections between participants and their employers and allowed warm handoffs to different resources across the state.

### 2.2. Participants

Participants (*n* = 80) were paraprofessionals in the BH workforce taking part in the BFT program. Participants were recruited in four cohorts (approximately one cohort per year) through professional networks and organizations, targeting community mental health centers and family resource centers. This sample includes those who participated in the BFT program, provided data, and provided consent for the data to be used for research purposes (72% of all those enrolled in the BFT program). Sample sizes may vary throughout the results section depending on the statistical tests being performed and the data collection methods being analyzed, given that not all participants who consented to be included in the study responded to every assessment survey, and not all respondents answered every question in each survey. To be eligible for Level I of the BFT program, participants needed to be employed in a paraprofessional role (regardless of previous educational attainment) and work directly with families affected by SUD or to have reported intention to be employed in such a role before the start of Level II on-the-job training. Demographic data is reported in Table 1. Some participants had earned higher degrees (often in unrelated fields, such as business or education) before changing careers. Earning a high school diploma or equivalent was the highest level of education for 51.3% of participants; 27.5% had a Bachelor’s degree, 16.3% had an Associate’s degree, and 5% had a Master’s degree. Throughout all four cohorts, most participants (81%) were female, white (83.5%), and non-Hispanic (94.7%). The mean age was 40.28 (*SD* = 10.78, range 19 to 67). Of note, most reported personal lived experience with substance misuse or mental health issues (57% and 65%, respectively). Of those, approximately 64% reported substance misuse during their childhood, and 62% reported mental health issues during their childhood. Many also reported being affected by the substance misuse of a loved one such as a parent, sibling, or partner/spouse (50%, 36.3%, and 41.3%, respectively).

**Figure 1:**
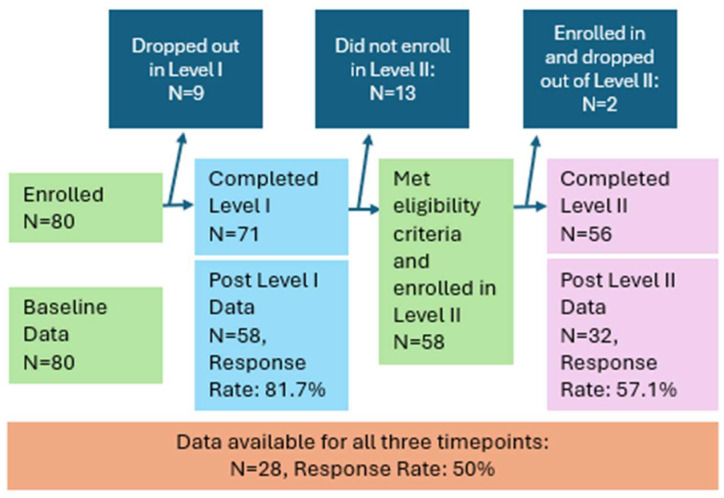
Flow chart of sample change over time.

Figure 1 shows the flow of participants through the three time points of the study. Since completion of the survey was considered research participation, and therefore, voluntary, not all trainees completed surveys, so data are not available for all participants. Further, continuing into Level II was based on stricter eligibility: participants had to have completed Level I successfully and be employed full-time in a paraprofessional role in a BH field—with an identified mentor who agreed to co-complete the competency portfolio—at the time of enrollment. About 18% (*n* = 13) of those who completed Level I were not eligible for Level II, mostly due to not being employed full-time in a position that met eligibility requirements.

### 2.3. Procedures

Assessments included an online survey administered via Qualtrics and distributed via email link at the following timepoints: (1) Baseline: before beginning the first course in Level I, (2) Post Level I: immediately after completing the final course in Level I and before beginning on-the-job training in Level II (approximately 7 months after baseline), and (3) Post Level II: after completion of Level II (approximately 18 months after baseline). The data from those who completed the training assessments were sent directly from Qualtrics to the evaluation team. Participants completed an initial survey as part of their orientation to the grant-funded program. While not required, completion of this baseline survey was strongly encouraged. Participants were asked separately for consent for their data to be used in this research project; this report includes only the data for those participants who provided consent. All research activities were approved by a university IRB. During Level II, participants tracked progress in several areas to demonstrate competence during their on-the-job training. The BFT team developed an Excel workbook into which participants and their mentors could collaboratively rate their level of competency in several domains (see below). A copy of the Excel workbook was shared with the program evaluators to analyze the data.

### 2.4. Measures

Measures for satisfaction, career intentions, knowledge, and confidence were developed for this research project based on the experience of the authors, including researchers with experience in program evaluation and the assessment of a medical training program. In some cases, we opted for face-valid, short or single-item measures to reduce burden on participants, based on research supporting the use of such measures for complex psychological constructs (Zimmerman et al., 2006).

#### 2.4.1. Satisfaction

After completion of Level I, participants were asked “how is the BFT program going for you overall?” on a 7-point scale with 1 being low and 7 being high. Then, participants were asked “Why did you choose this number?” and provided open-ended responses in their own words—these responses were treated as qualitative data. Participants were asked the same questions after the completion of Level II.

**Figure 2:**
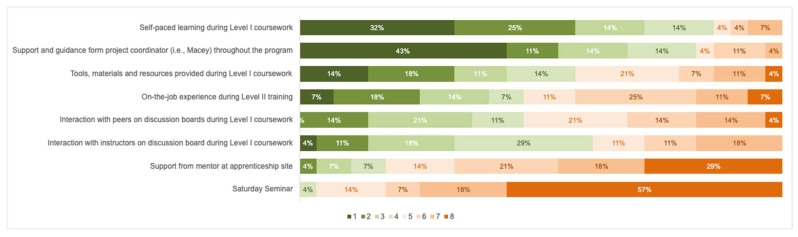
Results from Participants’ Ranking of Program Aspects after Level II.

After completion of Level II, participants were also asked “Please rank the following aspects of the BFT program in order of what contributed the most to your continued involvement and completion of the program. Rank the most important aspect #1, the second most important #2, etc.” Listed items reflected a range of program aspects from both Level I and Level II. Items were chosen based on anecdotal feedback from interactions with participants regarding different aspects of the program. The full list of eight items can be seen below, in Figure 2.

#### 2.4.2. Career Intentions

Career intention was measured at one time point, after completion of Level II. Participants finished this sentence as a measure of their career intention: “Due to my participation in the BFT program, I am…” (1) equally interested in continuing my education, (2) somewhat more interested in continuing my education, and (3) much more interested in continuing my education. Participants responded to the same item about “pursuing a career in behavioral health.”

#### 2.4.3. Effectiveness: Knowledge

Knowledge was measured by several questions on the content of each course, a total of 12 questions. These questions were developed by instructors and were based on a priori learning goals for each course. For each question, participants rated how much they agreed on a 5-point scale. Children, Youth, and Family Systems and Behavioral Health had three questions (e.g., “I can identify problems in family functioning, intergenerational patterns, and adverse childhood experiences, as a result of substance misuse”; baseline: α = 0.577, post-Level I: α = 0.853), The Four Domains of the Certified Recovery Support Worker had five (e.g., “I know what to do when I am concerned about youth impacted by family substance misuse”; baseline: α = 0.803, post-Level I: α = 0.851), and Enhanced Care Coordination had four (e.g., “I know how to access community and systems resources for children, youth and caregivers impacted by substance use disorders”; baseline: α = 0.739, post-Level I: α = 0.846). We averaged scores across all items within each course, which could range from 1 to 5, and then calculated the average score across participants for each course. Higher scores indicated a higher level of knowledge. Changes were measured by comparing scores from baseline to scores after completion of Level I.

#### 2.4.4. Effectiveness: Confidence

Confidence was measured with two questions. At all three time points, participants responded to the following question on a 5-point scale (strongly disagree to strongly agree), with higher scores indicating higher levels of confidence: “In general, I feel I understand children and youth impacted by family substance misuse.” After Level I and again at the end of the program (post-Level II), participants responded to the following question on a 5-point scale (much less confident to much more confident): “Compared to before starting the program, I would rate my current confidence in my ability to support youth impacted by family substance misuse as…”.

#### 2.4.5. Effectiveness: Perceived Competence

Perceived competence in on-the-job performance was measured during Level II. In collaboration with their mentors, participants rated themselves on several measures under five key competency domains in the Excel workbook. The competency assessment included a total of 113 items derived from an assessment used by the state Department of Labor. Scores were recorded before participants started Level II training and again after completing Level II. At both time points, trainees met with their mentors and rated their performance in each competency area using a 6-point scale that included scores of 0 (Learning), 1 (Understands), 2 (Developing), 3 (Competent), 4 (Skilled), and 5 (Master). A non-applicable option was available for items that did not pertain to the participant’s specific role. Mentors and participants were informed that Level 3 ratings were expected for each item within each competency for successful completion of an apprenticeship program, and Levels 4 and 5 should only be used to describe exceptional performance. The five domains of competency reflect specific predetermined skills:

- Domain 1: Enhanced care coordination—interdisciplinary teamwork, engagement, service development, and communication (44 items)
- Domain 2: Gathering, compiling, interpreting, and using data to guide decisions and service delivery (17 items)
- Domain 3: Employee performance relative to job-specific duties—administrative, scheduling, and routine tasks performed in the job (8 items)
- Domain 4: Mental processes—processing, planning, problem-solving, decision-making, and innovating activities performed with job-relevant information (37 items)
- Domain 5: Work output—physical activities, equipment/vehicles operated, and complex technical activities accomplished as job outputs (7 items)

### 2.5. Analytic Strategies

We used a single-group, repeated-measures design to measure change in target constructs over time. All participants (*n* = 80) completed the baseline assessment; 58 completed the post-Level I assessment, and 32 completed the post-Level II assessment (Figure 1). A total of 28 participants had data for all three time points; each test included only those participants with available data, meaning that the participant answered the survey *and* the question within the survey. We used listwise deletion/complete case analysis for each individual analysis; however, different analyses included data from different survey timepoints, and therefore, they have different sample sizes. When reporting statistical test results, we report the n that was used for each test.

Chi-square analyses were run to test differences in demographics between those who completed all three data timepoints (*n* = 28) and those who completed only two timepoints (or baseline only; *n* = 52), in order to assess whether this attrition is biasing the results at post-Level II. The only significant difference was for education: overall, those who had higher levels of education were *less* likely to complete data at all three timepoints (51.2% of high school graduates completed all three data timepoints, compared to 23.1% of those with an Associate’s degree, and 15.4% of those with a Bachelor’s degree or higher).

The data for assessments were downloaded from Qualtrics and analyzed using SPSS 28. Data from the four cohorts were collapsed into one dataset and treated as a single sample. We report descriptive statistics when relevant to study goals. When comparing across timepoints, data were tested using a repeated measures design. Scores based on five-point scales were treated as continuous variables for statistical tests. When data did not meet standards for normality (skewness and kurtosis > 1.0), we used nonparametric tests (e.g., Friedman’s Related Samples Analysis of Variance when comparing across the three time points and the Related Samples Wilcoxon Signed Ranks test for pairwise comparisons).

We also collected trainee perspectives about the effectiveness of the program by asking open-ended questions at the end of Level I and again at the end of Level II (“Why did you choose this number?” in response to general program satisfaction ratings). We used an analysis of common responses to this question to develop topics by identifying and grouping responses with similar language or terms that appeared most frequently.

## 3. Results

### 3.1. Satisfaction and Career Intention

At the end of Level I, 85.7% of participants rated their satisfaction with the program as 6 or 7 on a 7-point scale, with 7.2% responding with a score lower than 5. At the end of Level II, 86.7% of participants rated their satisfaction with the program as 6 or 7 on a 7-point scale, with no participants responding with a score lower than 5. Results from participants’ rankings of which aspects of the program were most effective for them are included in Figure 2. The number (1–8) corresponds to the participants’ ranking of that item, with a ranking of one indicating the item being of highest importance. Results imply that participants valued the role of the Project Coordinator (43% ranked as first choice) and the self-paced learning from Level I coursework (32% ranked as first choice) highest. The Saturday seminars and the role of the on-site mentor tended to be ranked lowest (with 57% and 29% ranking them lowest, respectively).

Regarding career intention, 6.3% of participants responded that they were *somewhat* more interested in continuing their education and 9.4% were somewhat more interested in pursuing a career in behavioral health due to their participation in the BFT program. More than half, 59.4%, of participants responded that they were *much* more interested in continuing their education and also pursuing a career in behavioral health.

### 3.2. Effectiveness: Knowledge, Confidence, and Perceived Competence

#### 3.2.1. Knowledge

**Figure 3:**
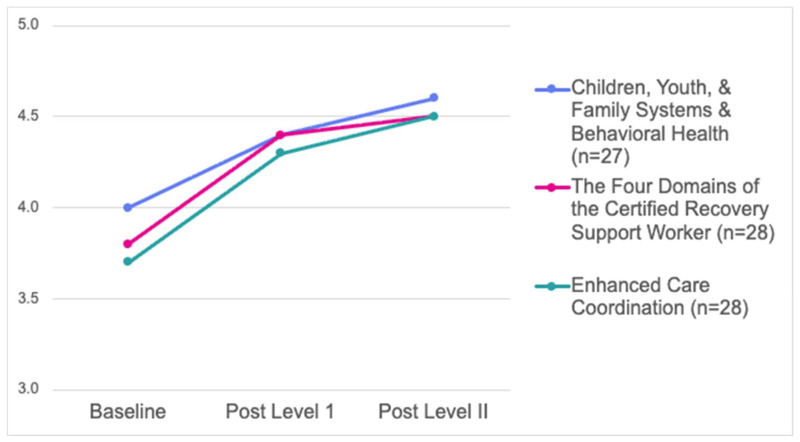
Change in knowledge by course across all time points.

Change in knowledge was tested using a series of Wilcoxon signed ranks tests comparing baseline and post-Level I timepoints for each course (*n* = 56). In the first course (Children, Youth, & Family Systems and Behavioral Health), the mean score increased from 4.07 (*SD* = 0.51) to 4.44 (*SD* = 0.47), *z* = 4.37, *p* < 0.001. In the second course (The Four Domains of the Certified Recovery Support Worker), the mean score increased from 3.73 (*SD* = 0.68) to 4.35 (*SD* = 0.46), *z* = 5.50, *p* < 0.001. In the third course (Enhanced Care Coordination), the mean score increased from 3.67 (*SD* = 0.64) to 4.31 (*SD* = 0.48), *z* = 5.41, *p* < 0.001. While we only tested the statistical significance of changes from baseline to post Level I, it is clear from Figure 3 that those changes were maintained through Level II. For data available at all three time points, results show a statistically significant increase in participants’ knowledge in all courses (see Figure 3).

#### 3.2.2. Confidence

Participant responses indicated an increase for the question “In general, I feel I understand children and youth impacted by family substance misuse.” At baseline, only 14.3% of participants reported the highest level of confidence in understanding the population they serve. This percentage increased after Level I training (45.7%) and continued to increase after Level II (54.3%). Results of a Friedman Related Samples Analysis of Variance test indicated an overall significant change in confidence, *X*^2^ = 20.10 (2), *p* < 0.001, *n* = 28. Post hoc Dunn’s tests with a Bonferroni adjustment revealed that the changes from baseline to post Level I were statistically significant (z = −2.81, *p* < 0.05), and the change from baseline to post Level II was significant (z = −3.01, *p* < 0.01). There was no significant change from post Level I to post Level II. Due to the change in sample size from post Level I to post Level II, we re-ran the test comparing change from baseline to post Level I using a Wilcoxon signed ranks test and the larger sample (*n* = 58); results again supported a significant change (*z* = 4.56, *p* < 0.001).

In response to the question “Compared to before starting the BFT program, I would rate my current confidence in my ability to support youth impacted by family substance misuse as…,” participants indicated improvements in confidence. At the end of Level I training, most participants selected either “more confident” (58.6%) or “much more confident” (34.5%). By the end of Level II, a higher proportion selected “much more confident”. More specifically, after Level II, 53.1% of participants responded “more confident” and 40.6% responded “much more confident.” Only 3.1% reported “about the same” and one respondent indicated a lower level of confidence by the end of Level II.

#### 3.2.3. Perceived Competence

Repeated measures *t*-tests comparing scores from before Level II to after Level II revealed that competency scores increased for all domains (see Table 2). These changes were statistically significant and reflected medium-to-large effect sizes (range 0.67–0.91). The largest effect was seen for Domain 3, employee performance relative to job-specific duties.

#### 3.2.4. Participant Perspectives

A review of common responses to the question asked after completion of Level I and Level II training, “How is this program going for you?” showed three consistent topics. The most frequent was the value of information and relevance to their work with families and youth. This was reflected in statements such as, “The program has helped me understand better the different approaches and techniques on how to help youths, caregivers and families impacted by substance abuse” and “I really enjoyed the classes and everything that I learned is able to help me daily in not just my work but in my personal life.” The second most mentioned topic was about how the program increased their *confidence* working with their population, reflected by comments such as, “I feel like I have learned a good deal of information and am more confident in my role and utilizing new information” and “Learned a lot, professional development, increased confidence, and also the financial incentive of the program was very helpful.” The third most frequent topic reflected in the comments was concerns about balancing work and home responsibilities with the training. This topic stood out with comments such as, “I’ve learned a lot but I did get overwhelmed between working full time and the amount of homework,” and “I’ve had some personal difficulties which have made time management, at times impossible.” However, many of the comments about the challenging workload were also mixed with high praise. For example, one trainee stated, “I love this program! It fits my learning style needs of not being a fast learner, needing lots of support and having the opportunity to work [on] things at my pace but also having a deadline for goals. It has really taught me a new perspective on schooling.”

## 4. Discussion

It is well known that many areas of the US are currently experiencing workforce shortages in the BH field, necessitating new and creative ways to bolster existing workers and encourage new ones to enter the field. The BFT program represents a novel approach that provides supplemental training to help paraprofessionals currently working in the BH field by providing academic and professional upskilling and training. Our program is based on the conceptual framework that certain aspects related to job satisfaction can be achieved by improving training—if BH workers feel more knowledgeable, confident, etc., and experience more achievement in their work, they will be more likely to persist in their career. Our results are encouraging, as there was support for both hypotheses. Results suggested that the program was well-received by participants, and subjective results provided preliminary evidence that the program was effective (as evidenced by improved knowledge, perceived competence, and confidence). Our results are also consistent with participation having a positive effect on career intentions. These results echo research that has employed paraprofessionals to address workforce challenges in schools and in the community—with practice standards for training, paraprofessionals can deliver high-quality and consistent services that are effective and meet community needs (Anvari et al., 2023; Bruns et al., 2025). Our study adds to this research by focusing on the effects that such trainings have on enhancing the perceived knowledge, skills, and tools for the paraprofessionals themselves, as well as their community. Finally, results provide preliminary support for using an apprenticeship model for BH trainees.

### 4.1. Changes in Knowledge, Confidence and Perceived Competence

Perhaps most encouraging was a change in confidence over the course of participation in the project. Our results indicated that coming into the study, few participants (14%) reported “strongly agree” to a statement related to how well they understood the population they serve. This is consistent with the lack of formal education and training found in the paraprofessional behavioral health workforce. This increased to 54% by the end of the program. These encouraging results are also echoed by the positive results regarding perceived competence, as rated by the participants and their mentors. This suggests that participants not only felt more confident but were actually better at their jobs, implying their confidence was well-founded. These results are reinforced by responses to the open-ended question about their experience with the program; participants emphasized how this program helped them to be better at their work. Most participants seemed to value and appreciate the opportunity to participate in this program.

Participants were able to take college-level courses that would not otherwise be available to them. The content of the courses was also relevant to their work. Notably, our courses cover the same topics that were highlighted by substance use peer support specialists in a qualitative study (Mumba et al., 2024). In that study, participants identified six themes that were considered to be educational needs for their work: (1) mental health and suicide prevention training, (2) diversity, equity, and inclusion training, (3) counseling skills training, (4) family systems approach to care training, (5) professionalism training, and (6) taking care of self—mind, soul, and body training (Mumba et al., 2024). Most of these (themes 1, 2, 3, and 4) were directly taught in Level 1 courses (our mental health module focused on trauma and substance use disorder but did not cover suicide prevention), and themes 5 and 6 were taught in Level 2, on-the-job training. The question of what content to include in such training is interesting. It could be argued that certain topics, such as intervention strategies and suicide prevention, are beyond the expectations of a paraprofessional/peer support role and should be handled by licensed clinicians. Indeed, certification of paraprofessionals has met resistance from state licensure boards for this reason (Areán et al., 2025). Given the population for this program, each of the courses was specifically designed for skill-building, provided a pathway to certification as a recovery support worker, and led trainees through specific skill-building activities that were applicable to their current positions. The courses also provided a theoretical background to help participants understand their clients and to deepen their understanding of their challenges, and hence, more empathy.

One of the unique aspects of the BFT program is the formal on-the-job training that participants received in the form of a state-sponsored apprenticeship program. Previous reports have suggested that employers benefit from apprenticeships in several ways—not only in increased productivity, but also in “indirect” benefits, such as improved workplace culture, pipelines, and loyalty (Marotta et al., 2022). In the BH field, which struggles with retention, these indirect effects are highly valuable. Our results provided support for this model, which is novel for the BH field. Participants expressed that they valued this aspect, along with the courses, and they continued to improve their learning and professional development, as evidenced by their improved perceived competence ratings. While these are subjective, they were rated jointly by the participants and mentors. This model was also popular among employers. Although participation led to a higher workload for the employer, they continued to participate in the BFT program. This report includes four cohorts; once an employer site was involved in the program, they tended to continue involvement year after year as long as they had an eligible employee.

### 4.2. Promotion and Retention Within Employer Site and Career Intentions

For a field with a reputation for high turnover and burnout among paraprofessionals (Areán et al., 2025), participants showed high levels of workforce retention. Most participants indicated interest in continuing their education and pursuing a career in behavioral health, which might include continuing in a paraprofessional role or pursuing a degree to advance further. This was an unanticipated benefit of the program, which is why we did not ask about career intentions in the baseline survey. During Level I, we noticed anecdotally that participant attitudes about pursuing a college degree evolved over the course of their participation—many were nervous about taking courses, but they valued the content, enjoyed learning, and discovered they were able to be successful. While not a direct outcome measured by our study, we hope that participation improved their perceived academic, “college-ready” skills to help them in this pursuit. It is worth noting that this was the first time that many participants had taken a college-level course; yet many reported an increased interest in pursuing further education after completion.

Our results indicate that the BFT program may be successful in addressing participant challenges related to completing formal training programs and retention in the workplace. Our approach was based on research indicating that retention and completion rates among disadvantaged community college students are higher if they receive academic, social, and behavioral health counseling and support (Holzer & Baum, 2017). As was clear in the open responses from participants, many struggled with time management and keeping up with the demands of the program. The BFT Project Coordinator addressed these challenges by forming relationships with and providing individualized planning and support to trainees. The Project Coordinator also worked with trainees on financial challenges, caregiver responsibilities, and natural support networks.

As was clear from the rankings of valued program aspects, participants indicated that the support they received from the Project Coordinator was critical to their success in the program. For example, if a participant was missing an important assignment and had not responded to the instructor’s inquiry, the Project Coordinator was often able to get in touch with that participant directly and encourage them to explain any barriers they may be experiencing to the instructor and request an extension, if appropriate. This is akin to academic coaching wherein the student learns time management and self-advocacy (Bettinger & Baker, 2014). It is important to note that while these participants were taking college-level courses, they did not have access to standard student support services such as tutoring, study groups, or academic advising, which are available at most institutions.

It is worth noting that participants with the lowest level of education (high school degree only) were *most* likely to continue in the program, including enrolling in Level II, not dropping out, and continuing to complete surveys. We consider this a testament to how much these participants valued their connection to the program. Simply put, the benefits from participation are probably most valuable to those with the lowest educational attainment, as they have the fewest options for career advancement.

When asked which elements of the program were most important for their success, 41% ranked “support and guidance from Project Coordinator” as their first choice for the program aspect that most contributed to their retention and success. Many of the trainees were meeting the demands of the program while working full-time, focusing on their own recovery, and taking care of children or other family members. While we were not able to directly measure why this role was perceived to be so important, we can speculate that the Project Coordinator was perceived to be an advocate or coach for participants, especially those who were struggling. The Project Coordinator would routinely be aware of life challenges (e.g., sickness, death in the family, etc.) well before the instructors. In this context, the results from the rankings (and open response feedback) suggest that this type of support was essential to their success. This implies that such training programs can be highly successful, but success may require persistent dedication to participant needs and challenges.

The two items ranked lowest were the support from mentors and the Saturday seminar. This result was somewhat surprising as mentorship is widely cited as an important aspect of job satisfaction in health fields (e.g., Heier & Nelson-Brantley, 2024). Participants in our study may have considered their mentors (most of whom were supervisors) as evaluative rather than instructive. Because interactions with mentors occurred in the professional setting, the research team had little involvement or control over the frequency and nature of these meetings, and the quality of the mentor relationship was not measured directly. It is also possible that mentors were less responsive or attentive to participants’ needs during on-the-job training in Level II, leading participants to perceive the mentors’ role to be less integral to their success. Finally, mentors in our study did not self-select for this role; in most cases they were selected by virtue of being the participant’s supervisor and may not have been intrinsically motivated to or understand how to provide true mentorship beyond supervisory duties. Future studies should investigate how mentor relationships in apprenticeship models can be better supported and also place more emphasis on studying the mentor-mentee relationship. Regarding the Saturday seminars, the research team received anecdotal feedback that these were difficult to attend, as the timing infringed on personal time. Hence, while the participants who attended them might have found them valuable, they might have been perceived as less valuable overall due to their effect on participants’ work–life balance.

### 4.3. Limitations and Future Directions

A strength of this study is our focus on the experiences and growth over time of the paraprofessionals who received this training. By focusing on the participants rather than their clients, we demonstrate the value of training and enhancing this level of the BH workforce. Our sample, though small, reflected a high degree of diversity in some areas, and most participants had previous mental health and SUD experience, highlighting the sample’s representativeness. This study also has several limitations.

First, most assessments were subjective, self-reports. While several of the measures (e.g., satisfaction, confidence) are highly subjective constructs, we recognize the limitations of self-reports for measures such as knowledge or competence. The subjectivity of these measures introduces the potential for bias. Unfortunately, it was not feasible to include objective measures of knowledge or job performance. It is important to note, however, that the measurement of perceived competence was completed jointly by the participant and their supervisor, reducing reliance on a single respondent. Further, while using data from client outcomes would have been valuable to assess the quality of training, this was not feasible as the BFT participants serve clients across many agencies statewide. Focusing on the experiences of the clients beyond those of the BFT participants was outside the scope of this project. However, we recommend future studies include objective measures of efficacy whenever appropriate.

Also, we were unable to include a control group to demonstrate that changes were due to the BFT program. While we recognize that including a control group would have been advantageous, this was not feasible due to the funding limitations and requirements. Lack of a control group means that it is possible that changes in target measures could have been due to other factors, such as maturation effects or simply the fact that participants knew they were being assessed. The time interval between measurement timepoints was rather long (at least seven months), reducing the likelihood of participants intentionally responding in ways that showed improvement.

Another factor that may have influenced participant responses is the stipends they received throughout the program. These stipends were provided at certain benchmarks and may have incentivized participants to continue their efforts in fulfilling the program’s requirements and their perceptions of the program in general. It is important to note, however, that very few participants mentioned the stipends as important factors in their open-ended responses about the program. Although the direct effects of the stipends were not measured, we acknowledge that they may have played a motivational role.

It is also important to acknowledge that some participants dropped out of the BFT program or were unable to continue into Level II. Some were unable to complete Level I successfully, indicating that even with the extensive support from Project Coordinators, some found the academic portion too difficult. Our sample comprised low-income, high-risk participants, many with a history of mental health or substance abuse challenges themselves. Many struggled with health problems, chaotic home lives, and a variety of life stressors that made participation difficult. Continuation into Level II was only an option if the participant had a full-time position in the BH field at the time of enrollment. While all in Level I had the *intention* to continue into this field in full-time work, not all did so successfully. Some may have been unable to continue into Level II due to personal reasons that were elucidated by their Level I experiences (e.g., time management, personal mental health problems, challenges with their own recovery journey, triggering nature of the work, etc.). This is a reminder that supplemental training programs—even when well-funded—add a strain to trainees’ lives that can pose barriers. It should also be acknowledged that this program was offered in 2020–2024, during and immediately after the COVID-19 pandemic and associated workforce disruption. It is possible that these decisions were influenced by the COVID-19 pandemic.

Finally, results are limited by the rather small sample size, made smaller by response rates. At each time point, our response rate was quite high: 100%, 81.7%, and 57.1% for baseline, post Level I, and post Level II respectively. This suggests that our sample was representative of the population of those enrolled in the BFT program. However, future studies should consider incentives for completing surveys, in addition to incentivizing program participation. Despite this limitation, the significant effects that we found across assessments, despite small sample size, are a testament to the robustness of the program’s potential.

Apprenticeship programs are underutilized in BH, and it is hoped that this study leads other similar developments, as well as increased standardization of competencies, training and supervision, and guidance for paraprofessionals in BH (Cooper et al., 2024; Bipartisan Policy Center, 2023). Other related concepts include collaborations with universities to offer course credit for similar trainings.

### 4.4. Conclusions

This study reports on the development and implementation of a novel, apprenticeship-based training program for BH trainees. Our results suggested that participation in the BFT program increased perceived knowledge, confidence, and perceived competence. This report provides preliminary evidence that providing high-quality training to paraprofessionals in the BH workforce may be an effective way to upskill the workforce, enhance workforce retention, and encourage ongoing educational attainment.

## Figures and Tables

**Table 1:** Demographic Characteristics of Participants at Baseline (*n* = 80).

	Baseline	
	*n*	*%*
Gender		
Male	14	17.7
Female	64	81.0
Trans/Nonbinary/Another	1	1.3
Highest level of education		
High school diploma or equivalent	41	51.3
Associates degree	13	16.3
Bachelor’s degree	22	27.5
Master’s degree or higher	4	5.0
Sexual orientation		
Choose not to disclose	1	1.3
Heterosexual	68	85.0
LGBTQ+	11	13.8
Race		
White	66	83.5
BIPOC	10	12.7
Multi-Racial	3	3.8
Ethnicity		
Non-Hispanic, Latino/a, or Spanish origin	71	94.7
Hispanic, Latino/a, or Spanish origin	4	5.3

**Table 2:** Competency Summary Results and Change over Level II training.

Domain	Mean at Start of Level II	Mean After Level II	Score Change	*t*	df	Two-Sided *p*	Cohen’s D
1	2.669	4.102	1.434	−14.267	45	<0.001	0.67
2	2.438	3.807	1.369	−11.253	45	<0.001	0.84
3	2.659	4.120	1.461	−10.555	45	<0.001	0.91
4	2.667	4.076	1.409	−12.497	45	<0.001	0.77
5	3.094	4.311	1.217	−10.454	44	<0.001	0.76

Note: *n* = 46.

## Data Availability

The datasets presented in this article are not readily available because the data are part of an ongoing study. Requests to access the datasets should be directed to the corresponding author.

## Abbreviations

The following abbreviations are used in this manuscript:

BH	Behavioral health
SUD	Substance use disorder
DOL	Department of Labor
HRSA	Health Resources Services Administrations
BFT	Building Futures Together

## References

[B1-behavsci-16-00441] Anvari M. S., Hampton T., Tong M. P., Kahn G., Triemstra J. D., Magidson J. F., Felton J. W. (2023). Behavioral activation disseminated by non-mental health professionals, paraprofessionals, and peers: A systematic review. Behavior Therapy.

[B2-behavsci-16-00441] Areán P. A., O’Connor S., Sherrill J. (2025). The promise and perils of using peers and other paraprofessionals as mental health service professionals. JAMA Psychiatry.

[B3-behavsci-16-00441] Ashrafioun L., Dambra C. M., Blondell R. D. (2011). Parental prescription opioid abuse and the impact on children. The American Journal of Drug and Alcohol Abuse.

[B4-behavsci-16-00441] Ballout S. (2025). Trauma, mental health workforce shortages, and health equity: A crisis in public health. International Journal of Environmental Research and Public Health.

[B5-behavsci-16-00441] Barnett M. L., Gonzalez A., Miranda J., Chavira D. A., Lau A. S. (2018). Mobilizing community health workers to address mental health disparities for underserved populations: A systematic review. Administration and Policy in Mental Health and Mental Health Services Research.

[B6-behavsci-16-00441] Beck A. J., Manderscheid R. W., Buerhaus P. (2018). The future of the behavioral health workforce: Optimism and opportunity. American Journal of Preventive Medicine.

[B7-behavsci-16-00441] Bettinger E. P., Baker R. B. (2014). The effects of student coaching: An evaluation of a randomized experiment in student advising. Educational Evaluation and Policy Analysis.

[B8-behavsci-16-00441] Bina R., Harnek Hall D. M., Mollette A., Smith-Osborne A., Yum J., Sowbel L., Jani J. (2008). Substance abuse training and perceived knowledge: Predictors of perceived preparedness to work in substance abuse. Journal of Social Work Education.

[B9-behavsci-16-00441] Bipartisan Policy Center (2023). Recommendations for building workforce capacity by increasing behavioral health support specialists. Filling the gaps in the behavioral health workforce.

[B10-behavsci-16-00441] Bruns E. J., Ehde C., Gaias L. M., Kebede B., McWherter C., Wick E. (2025). Behavioral health student assistance programs: Leveraging non-traditional mental health providers to address workforce shortages and mitigate the youth mental health crisis. Administration and Policy in Mental Health and Mental Health Services Research.

[B11-behavsci-16-00441] Butrica B. A., Jones E., Rosenberg L., Sattar S., Sotelo V. (2023). A review of the literature on registered apprenticeships: Evaluating registered apprenticeship initiatives.

[B12-behavsci-16-00441] Cooper R. E., Saunders K. R. K., Greenburgh A., Shah P., Appleton R., Machin K., Jeynes T., Barnett P., Allan S. M., Griffiths J., Stuart R., Mitchell L., Chipp B., Jeffreys S., Lloyd-Evans B., Simpson A., Johnson S. (2024). The effectiveness, implementation, and experiences of peer support approaches for mental health: A systematic umbrella review. BMC Medicine.

[B13-behavsci-16-00441] Daley D. C. (2013). Family and social aspects of substance use disorders and treatment. Journal of Food and Drug Analysis.

[B14-behavsci-16-00441] de Amesti J., Claro S. (2021). Effects of apprenticeship on the short-term educational outcomes of vocational high-school students. Journal of Research on Educational Effectiveness.

[B15-behavsci-16-00441] Fill M.-M. A., Miller A. M., Wilkinson R. H., Warren M. D., Dunn J. R., Schaffner W., Jones T. F. (2018). Educational disabilities among children born with neonatal abstinence syndrome. Pediatrics.

[B16-behavsci-16-00441] Gagne C. A., Finch W. L., Myrick K. J., Davis L. M. (2018). Peer workers in the behavioral and integrated health workforce: Opportunities and future directions. American Journal of Preventive Medicine.

[B17-behavsci-16-00441] Heath Resources Services Administration (2020). Opioid-impacted family support program.

[B18-behavsci-16-00441] Hedegaard H., Warner M., Miniño A. M. (2017). Drug overdose deaths in the United States, 1999–2016.

[B19-behavsci-16-00441] Heier C., Nelson-Brantley H. (2024). Nurse faculty job satisfaction: A concept analysis. The Journal of Continuing Education in Nursing.

[B20-behavsci-16-00441] Holzer H. J., Baum S. (2017). Making college work: Pathways to success for disadvantaged students.

[B21-behavsci-16-00441] Kanzler K. E., Kunik M. E., Aycock C. A. (2024). Increasing access to behavioral health care: Examples of task shifting in two U.S. government health care systems. Families, Systems, & Health.

[B22-behavsci-16-00441] Kuehn D., Mills De La Rosa S., Lerman R., Hollenbeck K. (2022). Do employers earn positive returns to investments in apprenticeship? Evidence from registered programs under the American apprenticeship initiative.

[B23-behavsci-16-00441] Lowder E. M., Foudray C. M. A., Thai M., Freund R., Lane M. (2025). Multiprogram perspectives on the peer recovery specialist role, opportunities, and challenges. Psychiatric Rehabilitation Journal.

[B24-behavsci-16-00441] Manzo J., Manzo F., Bruno R. (2019). The Impact of construction apprenticeship programs in Minnesota: A return-on-investment analysis.

[B25-behavsci-16-00441] Marotta J., Lerman R., Kuehn D., San Miguel M. (2022). Beyond productivity: How employers gain more from apprenticeship: Findings from the American apprenticeship initiative evaluation.

[B26-behavsci-16-00441] Mian N. D., Glutting J. H. (2025). Leaks in the workforce pipeline: Understanding barriers to pursuing mental health careers among undergraduate psychology students. Teaching of Psychology.

[B27-behavsci-16-00441] Mumba M. N., Sweeney A., Jennings C., Matthews J., Andrabi M., Hall J., Benstead H. (2024). Perceived educational needs of substance use peer support specialists: A qualitative study. Community Mental Health Journal.

[B28-behavsci-16-00441] Paris M., Hoge M. A. (2010). Burnout in the mental health workforce: A review. The Journal of Behavioral Health Services & Research.

[B29-behavsci-16-00441] Reed D., Liu A. Y.-H., Kleinman R., Mastri A., Reed D., Sattar S., Ziegler J. (2012). An effectiveness assessment and cost-benefit analysis of registered apprenticeship in 10 states.

[B30-behavsci-16-00441] Satinsky E. N., Doran K., Felton J. W., Kleinman M., Dean D., Magidson J. F. (2020). Adapting a peer recovery coach-delivered behavioral activation intervention for problematic substance use in a medically underserved community in Baltimore City. PLoS ONE.

[B31-behavsci-16-00441] Wilcox M. M., Barbaro-Kukade L., Pietrantonio K. R., Franks D. N., Davis B. L. (2021). It takes money to make money: Inequity in psychology graduate student borrowing and financial stressors. Training and Education in Professional Psychology.

[B32-behavsci-16-00441] Winstanley E. L., Stover A. N. (2019). The impact of the opioid epidemic on children and adolescents. Clinical Therapeutics.

[B33-behavsci-16-00441] Yen E., Davis J. M. (2022). The immediate and long-term effects of prenatal opioid exposure. Frontiers in Pediatrics.

[B34-behavsci-16-00441] Zimmerman M., Ruggero C. J., Chelminski I., Young D., Posternak M. A., Friedman M., Boerescu D., Attiullah N. (2006). Developing brief scales for use in clinical practice: The reliability and validity of single-item self-report measures of depression symptom severity, psychosocial impairment due to depression, and quality of life. The Journal of Clinical Psychiatry.

